# Crystal structure of (±)-(4*RS*,5*RS*,7*SR*)-4-[(1*RS*,2*RS*,3*RS*,6*RS*)-3-benzo­yloxy-2-(2-hy­droxy­ethyl)-6-meth­oxy­meth­oxy-2-methyl­cyclo­hex­yl]-8,10,10-trimethyl-2-oxo-1,3-dioxa­spiro­[4.5]dec-8-en-7-yl benzoate benzene monosolvate

**DOI:** 10.1107/S2056989014026048

**Published:** 2015-01-01

**Authors:** Takeshi Oishi, Yuu Yamaguchi, Keisuke Fukaya, Tomoya Sugai, Ami Watanabe, Takaaki Sato, Noritaka Chida

**Affiliations:** a School of Medicine, Keio University, Hiyoshi 4-1-1, Kohoku-ku, Yokohama 223-8521, Japan; b Department of Applied Chemistry, Faculty of Science and Technology, Keio University, Hiyoshi 3-14-1, Kohoku-ku, Yokohama 223-8522, Japan

**Keywords:** crystal structure, hydrogen bonds, taxane skeleton, paclitaxel

## Abstract

In the title compound, C_36_H_44_O_10_·C_6_H_6_, the dioxolane ring adopts an envelope conformation with the C atom bonded to the H atom as the flap, while the cyclo­hexene and cyclo­hexane rings are in half-chair and chair conformations, respectively. In the crystal, a pair of O—H⋯O hydrogen bonds with an *R*_2_^2^(26) graph-set motif connect the benzoate mol­ecules into an inversion dimer. The dimers are linked by a weak C—H⋯O inter­action into a tape structure along [01-1]. The benzene mol­ecule links the tapes through C—H⋯O and C—H⋯π inter­actions, forming a sheet parallel to (100).

## Abstract 2

In the title compound, the ring conformations of the tricycles are in an envelope, a half-chair and a chair. In the crystal, inter­molecular O—H⋯O and C—H⋯O hydrogen bonds and C—H⋯π inter­actions link the mol­ecules into a three-dimensional architecture.

## Chemical context   

**Figure 1:**
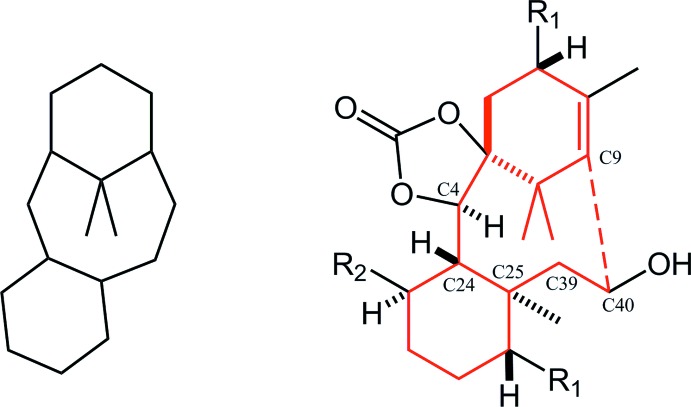
Left: the structure of the tri­cyclo­[9.3.1.0^3,8^]penta­decane (taxane) skeleton. Right: the title compound. Red lines indicate the taxane skeleton with the expected bond (red dashed line). *R*_1_ = –OC(=O)Ph, *R*_2_ = –OCH_2_OCH_3_.

Paclitaxel is a well-known natural diterpenoid containing a taxane framework (tri­cyclo­[9.3.1.0^3,8^]penta­decane; Fig. 1 ▸), with potent anti­tumor activity (Wall & Wani, 1995 ▸). This unique and complicated structure has attracted significant inter­est, and a large number of synthetic studies have been reported. In these researches, whereas some structure data *after* cyclization into taxane or taxoid derivatives are available (§ 4), precursors *just before* cyclization are very few. The title compound has been obtained in our synthetic study of paclitaxel as a cyclization precursor to build the taxane skeleton (Fukaya *et al.*, 2014 ▸).
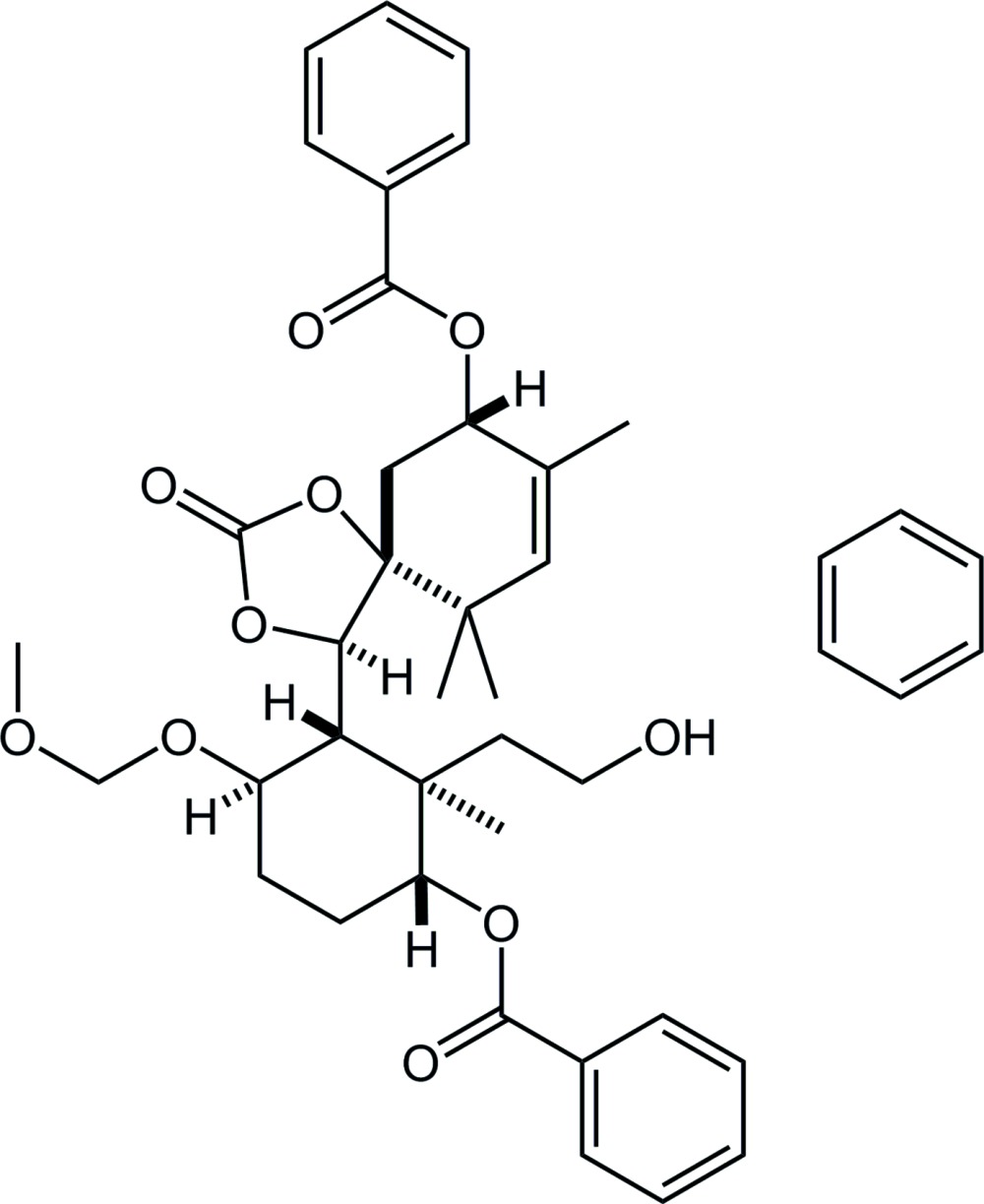


## Structural commentary   

**Figure 2:**
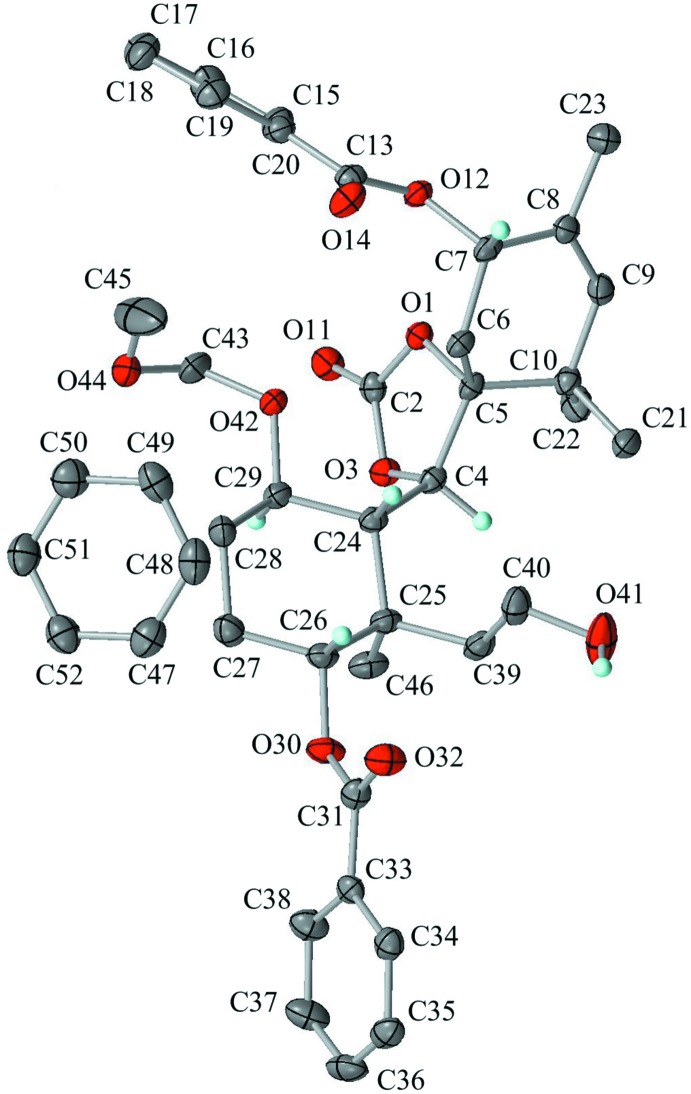
The asymmetric unit of the title compound with atom labelling. Displacement ellipsoids are drawn at the 50% probability level. Only H atoms connected to O and chiral C atoms are shown for clarity.

**Figure 3:**
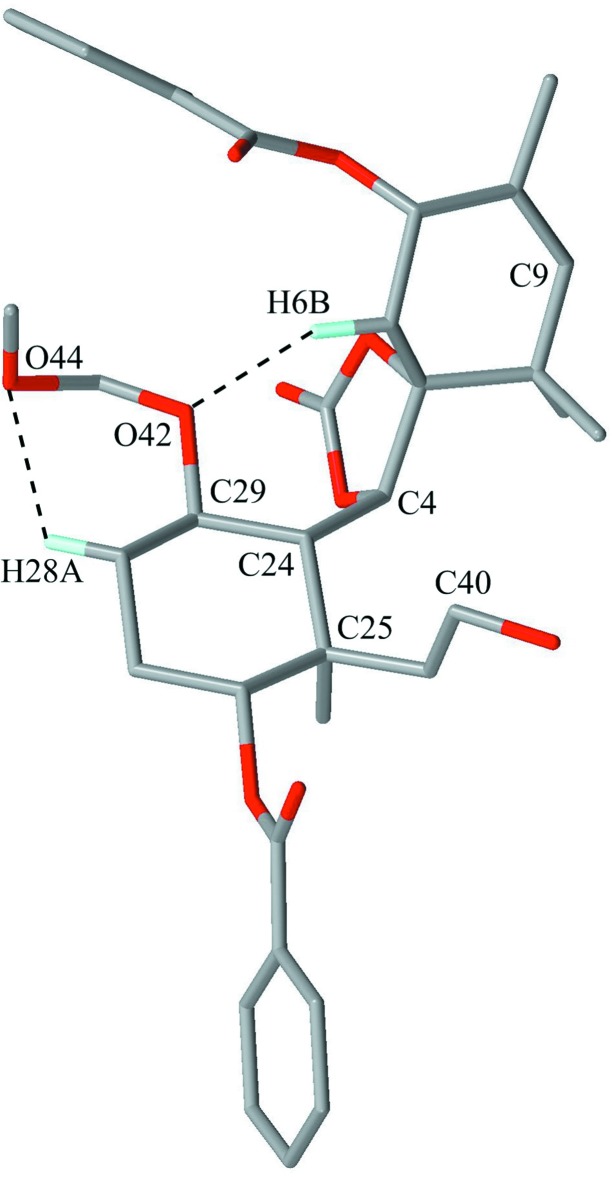
The mol­ecular conformation indicating the intra­molecular C—H⋯O inter­actions with dashed lines. Only H atoms involved in hydrogen bonds are shown for clarity. The benzene solvent mol­ecule has been omitted.

The mol­ecular structure of the title compound is shown in Fig. 2 ▸. The dioxolane ring (O1/C2/O3/C4/C5) is in an envelope conformation with puckering parameters of *Q*(2) = 0.165 (2) Å and ϕ(2) = 114.5 (6)°. The flap atom C4 deviates from the mean plane of other atoms by 0.270 (3) Å. The cyclo­hexene ring (C5–C10), which is spiro-fused to the dioxolane ring, is in a half-chair conformation with puckering parameters of *Q* = 0.469 (2) Å, θ = 127.5 (2)°, ϕ(2) = 197.2 (3)°, *Q*(2) = 0.372 (2) Å and *Q*(3) = −0.285 (2) Å. Atoms C5 and C6 deviate from the mean plane of the other atoms by −0.493 (4) and 0.212 (4) Å, respectively. The cyclo­hexane ring (C24–C29) is in a chair conformation with puckering parameters *Q* = 0.587 (2) Å, θ = 4.6 (2)°, ϕ = 246 (3)°, Q(2) = 0.042 (2) Å and Q(3) = 0.585 (2) Å. The large substituents (C24—C4, C25—C39, C26—O30 and C29—O42) are in the equatorial positions. The meth­oxy­meth­oxy group (O42/C43/O44/C45) shows a helical form with torsion angles of 76.5 (3)° for C29—O42—C43—O44 and 64.8 (3)° for O42—C43—O44—C45 held by weak intra­molecular C—H⋯O inter­actions (Fig. 3 ▸, Table 1 ▸). The atom pairs which may be connected by cyclization into a taxane framework are C9 and C40 (Figs. 1 ▸ and 3 ▸) with their distance being 5.831 (3) Å in the present conformation. They are expected to approach each other by rotation about the C4–C24, C25–C39 and C39–C40 bonds.

## Supra­molecular features   

**Figure 4:**
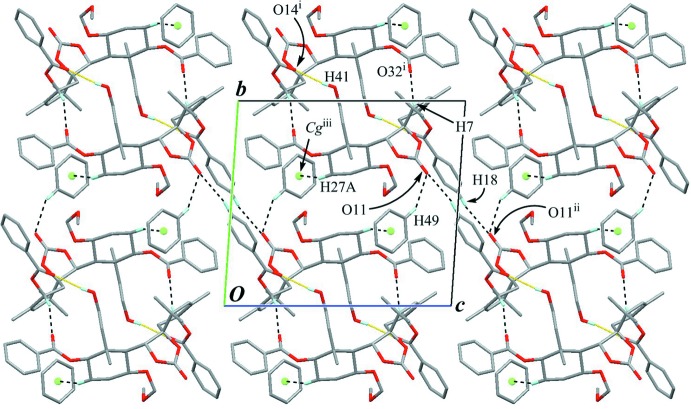
The crystal packing viewed along the *a* axis. Dotted yellow lines indicate the inter­molecular O—H⋯O hydrogen bonds which form the inversion dimers. Black dashed lines indicate the inter­molecular C—H⋯O and C—H⋯π inter­actions. *Cg* is the centroid of the benzene solvent mol­ecule. Only H atoms involved in hydrogen bonds are shown for clarity. [Symmetry codes: (i) −*x* + 1, −*y* + 2, −*z* + 1; (ii) −*x* + 1, −*y* + 1, −*z* + 2; (iii) −*x* + 1, −*y* + 1, −*z* + 1.]

The crystal packing is stabilized by a pair of inter­molecular O—H⋯O hydrogen bonds (O41—H41⋯O14^i^; Table 1 ▸) with an 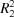
(26) graph-set motif, forming an inversion dimer (Fig. 4 ▸). In the dimer, a pair of C—H⋯O hydrogen bonds (C7—H7⋯O32^i^; Table 1 ▸) are also observed. The dimers are further linked by a weak inter­molecular C—H⋯O hydrogen bond (C18—H18⋯O11^ii^; Table 1 ▸) into a tape along [01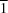]. The benzene mol­ecule links adjacent tapes through C—H⋯O and C—H⋯π inter­actions (C49—H49⋯O11 and C27—H27*A*⋯*Cg* ^iii^; Table 1 ▸), forming a sheet parallel to (100).

## Database survey   

**Figure 5:**
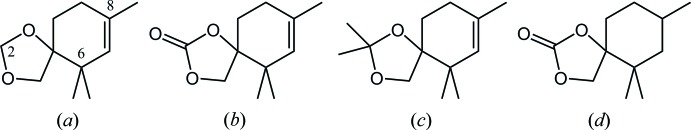
Core substructures for database survey; (*a*) 6,6,8-trimethyl-1,3-dioxa­spiro­[4.5]dec-7-ene, (*b*) its 2-one derivative, (*c*) the 2,2-dimethyl derivative and (*d*) 6,6,8-trimethyl-1,3-dioxa­spiro­[4.5]decan-2-one.

In the Cambridge Structural Database (CSD, Version 5.35, November 2013; Groom & Allen, 2014 ▸), four compounds possessing a core of 6,6,8-trimethyl-1,3-dioxa­spiro­[4.5]dec-7-ene are found (Fig. 5 ▸). These include its derivatives with 2-one (PUQLAO; Nishizawa *et al.*, 1998 ▸) and 2,2-dimethyl (NEGBOQ; Poujol *et al.*, 1997 ▸) substitutes. Another tetra­cyclic taxoid (ILIQUP; Ohba *et al.*, 2003 ▸) with a core of 6,6,8-trimethyl-1,3-dioxa­spiro­[4.5]decan-2-one, obtained in our previous study, is closely related to the title compound. Only one crystalline compound just before cyclization is found in the literature (Nicolaou *et al.*, 1995 ▸), however it is not registered in the CSD.

## Synthesis and crystallization   

The title compound was obtained in a synthetic study on paclitaxel. The cyclo­hexene unit (C5–C10) was provided according to the reported procedure (Nicolaou *et al.*, 1995 ▸), and coupled with the substituted cyclo­hexane unit (C24–C29) synthesized from 3-methyl­anisole (Fukaya *et al.*, 2014 ▸) by a Shapiro reaction (Nicolaou *et al.*, 1995 ▸). Further manipulation of the functional groups afforded the title compound, which was purified by silica gel column chromatography. Colorless crystals were grown from a benzene solution under a pentane-saturated atmosphere by slow evaporation at ambient temperature.

## Refinement   

Crystal data, data collection and structure refinement details are summarized in Table 2 ▸. C-bound H atoms were positioned geometrically with C—H = 0.95–1.00 Å, and constrained to ride on their parent atoms with *U*_iso_(H) = 1.2*U*_eq_(C) or 1.5*U*_eq_(methyl C). The H atom of hy­droxy group (O41) was placed guided by difference maps and then treated as riding, with O—H = 0.84 Å and with *U*_iso_(H) = 1.5*U*_eq_(O). 13 problematic reflections were omitted from the final refinement.

## Supplementary Material

Crystal structure: contains datablock(s) global, I. DOI: 10.1107/S2056989014026048/is5382sup1.cif

Structure factors: contains datablock(s) I. DOI: 10.1107/S2056989014026048/is5382Isup2.hkl

Supporting information file. DOI: 10.1107/S2056989014026048/is5382Isup3.cml

CCDC reference: 1036428

Additional supporting information:  crystallographic information; 3D view; checkCIF report

## Figures and Tables

**Table 1:** Hydrogen-bond geometry (, ) *Cg* is the centroid of the C47C52 ring.

*D*H*A*	*D*H	H*A*	*D* *A*	*D*H*A*
C6H6*B*O42	0.99	2.32	3.095(2)	135
C28H28*A*O44	0.99	2.37	2.989(3)	120
O41H41O14^i^	0.84	2.06	2.888(2)	170
C7H7O32^i^	1.00	2.34	3.269(2)	155
C18H18O11^ii^	0.95	2.53	3.465(2)	168
C49H49O11	0.95	2.46	3.300(3)	147
C27H27*A* *Cg* ^iii^	0.95	2.64	3.514(2)	147

Symmetry codes: (i) 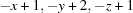; (ii) 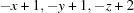; (iii) 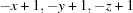.

**Table 2:** Experimental details

Crystal data	
Chemical formula	C_36_H_44_O_10_C_6_H_6_
*M*_r_	714.82
Crystal system, space group	Triclinic, *P* 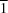
Temperature (K)	90
*a*, *b*, *c* ()	9.6397(6), 13.6008(8), 15.0461(10)
, , ()	83.6966(19), 77.488(2), 77.9768(18)
*V* (^3^)	1879.2(2)
*Z*	2
Radiation type	Mo *K*
(mm^1^)	0.09
Crystal size (mm)	0.50 0.37 0.19

Data collection	
Diffractometer	Bruker D8 Venture
Absorption correction	Multi-scan (*SADABS*; Bruker, 2013 ▸)
*T*_min_, *T*_max_	0.96, 0.98
No. of measured, independent and observed [*I* > 2(*I*)] reflections	25389, 6526, 5180
*R*_int_	0.043
(sin /)_max_ (^1^)	0.595

Refinement	
*R*[*F* ^2^ > 2(*F* ^2^)], *wR*(*F* ^2^), *S*	0.042, 0.151, 0.93
No. of reflections	6526
No. of parameters	475
H-atom treatment	H-atom parameters constrained
_max_, _min_ (e ^3^)	0.25, 0.25

Computer programs: *APEX2* and *SAINT* (Bruker, 2013 ▸), *SHELXS2013* and *SHELXL2014* (Sheldrick, 2008 ▸), *Mercury* (Macrae *et al.*, 2006 ▸), *publCIF* (Westrip, 2010 ▸) and *PLATON* (Spek, 2009 ▸).

## supplementary crystallographic information

### Crystal data

**Table:**

C_36_H_44_O_10_·C_6_H_6_	*F*(000) = 764
*M**_r_* = 714.82	*D*_x_ = 1.263 Mg m^−^^3^
Triclinic, *P*1	Melting point: 459.2 K
*a* = 9.6397 (6) Å	Mo *K*α radiation, λ = 0.71073 Å
*b* = 13.6008 (8) Å	Cell parameters from 9980 reflections
*c* = 15.0461 (10) Å	θ = 2.2–25.1°
α = 83.6966 (19)°	µ = 0.09 mm^−^^1^
β = 77.488 (2)°	*T* = 90 K
γ = 77.9768 (18)°	Plate, colorless
*V* = 1879.2 (2) Å^3^	0.50 × 0.37 × 0.19 mm
*Z* = 2	

### Data collection

**Table:**

Bruker D8 Venture diffractometer	6526 independent reflections
Radiation source: fine-focus sealed tube	5180 reflections with *I* > 2σ(*I*)
Multilayered confocal mirror monochromator	*R*_int_ = 0.043
Detector resolution: 8.333 pixels mm^-1^	θ_max_ = 25.0°, θ_min_ = 2.2°
φ and ω scans	*h* = −11→11
Absorption correction: multi-scan (*SADABS*; Bruker, 2013)	*k* = −16→16
*T*_min_ = 0.96, *T*_max_ = 0.98	*l* = −17→17
25389 measured reflections	

### Refinement

**Table:**

Refinement on *F*^2^	Primary atom site location: structure-invariant direct methods
Least-squares matrix: full	Secondary atom site location: difference Fourier map
*R*[*F*^2^ > 2σ(*F*^2^)] = 0.042	Hydrogen site location: inferred from neighbouring sites
*wR*(*F*^2^) = 0.151	H-atom parameters constrained
*S* = 0.93	*w* = 1/[σ^2^(*F*_o_^2^) + (0.0996*P*)^2^ + 1.0739*P*] where *P* = (*F*_o_^2^ + 2*F*_c_^2^)/3
6526 reflections	(Δ/σ)_max_ = 0.023
475 parameters	Δρ_max_ = 0.25 e Å^−^^3^
0 restraints	Δρ_min_ = −0.25 e Å^−^^3^

### Special details

**Table:**

Experimental. Recrystallization from benzene, toluene, chloroform, dichloromethane, diethyl ether, tetrahydrofuran, ethyl acetate, acetonitrile and methanol solutions under the air were failed. These solutions under hexane atmosphere also gave unsatisfactory results. Only the condition mentioned above has been quite effective to afford the single crystals suitable for X-ray analysis.; *M*.p. 458.7–459.2 K (not corrected); IR (film): 3524, 2945, 2890, 1790, 1715, 1274, 714 cm^-1^; ^1^H NMR (500 MHz, CDCl_3_): *δ* (p.p.m.) 8.13–8.10 (m, 2H), 8.03–7.99 (m, 2H), 7.60–7.53 (m, 2H), 7.48–7.42 (m, 4H), 5.56 (d, *J* = 6.0 Hz, 1H), 5.41 (t, *J* = 0.9 Hz, 1H), 5.09 (dd, *J* = 11.5, 4.6 Hz, 1H), 4.84 (s, 1H), 4.49 (d, *J* = 7.7 Hz, 1H), 4.13 (d, *J* = 7.7 Hz, 1H), 3.76–3.68 (m, 2H), 3.63 (ddd, *J* = 10.6, 7.2, 7.2 Hz, 1H), 3.08 (d, *J* = 14.9 Hz, 1H), 2.66 (s, 3H), 2.40 (dddd, *J* = 12.9, 4.3, 4.0, 4.0 Hz, 1H), 2.34 (d, *J* = 10.3 Hz, 1H), 2.29 (dd, *J* = 15.5, 6.3 Hz, 1H), 1.93 (dddd, *J* = 12.9, 4.3, 4.3, 4.0 Hz, 1H), 1.75 (d, *J* = 0.9 Hz, 3H), 1.73–1.60 (m, 3H), 1.59–1.45 (m, 2H), 1.22 (s, 3H), 1.20 (s, 3H), 1.11 (s, 3H); ^13^C NMR (125 MHz, CDCl_3_): *δ* (p.p.m.) 166.4 (C), 166.0 (C), 155.2 (C), 135.1 (CH), 133.4 (CH), 133.3 (CH), 130.5 (C), 130.2 (C), 130.0 (CH), 129.7 (CH), 129.0 (C), 128.7 (CH), 128.7 (CH), 97.9 (CH_2_), 87.1 (C), 76.9 (CH), 76.5 (CH), 75.1 (CH), 68.7 (CH), 58.3 (CH_2_), 54.7 (CH_3_), 45.9 (CH), 42.0 (C), 41.2 (C), 38.1 (CH_2_), 31.5 (CH_2_), 30.6 (CH_2_), 25.7 (CH_3_), 25.1 (CH_2_), 22.3 (CH_3_), 20.2 (CH_3_), 16.8 (CH_3_); HRMS (ESI): *m/z* calcd for C_36_H_44_O_10_Na^+^ [*M*+Na]^+^ 659.2832, found 659.2836.
Geometry. All e.s.d.'s (except the e.s.d. in the dihedral angle between two l.s. planes) are estimated using the full covariance matrix. The cell e.s.d.'s are taken into account individually in the estimation of e.s.d.'s in distances, angles and torsion angles; correlations between e.s.d.'s in cell parameters are only used when they are defined by crystal symmetry. An approximate (isotropic) treatment of cell e.s.d.'s is used for estimating e.s.d.'s involving l.s. planes.
Refinement. Refinement of *F*^2^ against ALL reflections. The weighted *R*-factor *wR* and goodness of fit *S* are based on *F*^2^, conventional *R*-factors *R* are based on *F*, with *F* set to zero for negative *F*^2^. The threshold expression of *F*^2^ > σ(*F*^2^) is used only for calculating *R*-factors(gt) *etc*. and is not relevant to the choice of reflections for refinement. *R*-factors based on *F*^2^ are statistically about twice as large as those based on *F*, and *R*- factors based on ALL data will be even larger.Problematic 13 reflections with |*I*(obs)-*I*(calc)|/σW(*I*) greater than 10 (–8 –2 1, –8 –2 2, –8 –1 2, –7 –4 3, –8 –3 6, 3 11 7, 3 10 8, 0 9 9, 2 10 9, 2 8 10, 4 9 10, 2 7 11, 3 8 11) have been omitted in the final refinement.

### Fractional atomic coordinates and isotropic or equivalent isotropic displacement parameters (Å^2^)

**Table:**

	*x*	*y*	*z*	*U*_iso_*/*U*_eq_	
O1	0.25606 (14)	0.79849 (9)	0.81366 (8)	0.0191 (3)	
C2	0.2032 (2)	0.71963 (14)	0.79948 (13)	0.0201 (4)	
O3	0.16170 (14)	0.73096 (9)	0.71879 (9)	0.0206 (3)	
C4	0.2098 (2)	0.81686 (14)	0.66496 (13)	0.0185 (4)	
H4	0.1247	0.8597	0.6428	0.022*	
C5	0.2481 (2)	0.87501 (14)	0.73783 (12)	0.0181 (4)	
C6	0.3931 (2)	0.90852 (14)	0.71464 (13)	0.0194 (4)	
H6A	0.3918	0.9613	0.6638	0.023*	
H6B	0.4698	0.8506	0.6934	0.023*	
C7	0.4298 (2)	0.94956 (14)	0.79519 (13)	0.0204 (4)	
H7	0.5042	0.9925	0.7716	0.024*	
C8	0.3023 (2)	1.01014 (14)	0.85459 (13)	0.0204 (4)	
C9	0.1692 (2)	1.01625 (14)	0.84154 (13)	0.0219 (4)	
H9	0.094	1.0582	0.8799	0.026*	
C10	0.1244 (2)	0.96338 (14)	0.77197 (13)	0.0206 (4)	
O11	0.19139 (15)	0.64864 (10)	0.85222 (9)	0.0258 (3)	
O12	0.48877 (14)	0.86671 (9)	0.85533 (9)	0.0206 (3)	
C13	0.6265 (2)	0.82130 (14)	0.82802 (13)	0.0204 (4)	
O14	0.70642 (15)	0.84907 (11)	0.76027 (10)	0.0279 (3)	
C15	0.6684 (2)	0.73187 (15)	0.88941 (13)	0.0221 (4)	
C16	0.8142 (2)	0.68716 (16)	0.88007 (14)	0.0264 (5)	
H16	0.8853	0.7165	0.8378	0.032*	
C17	0.8556 (2)	0.60012 (16)	0.93234 (16)	0.0324 (5)	
H17	0.955	0.5699	0.9263	0.039*	
C18	0.7518 (3)	0.55705 (16)	0.99346 (15)	0.0328 (5)	
H18	0.7799	0.4965	1.0285	0.039*	
C19	0.6074 (3)	0.60200 (16)	1.00363 (15)	0.0321 (5)	
H19	0.5367	0.5729	1.0465	0.039*	
C20	0.5651 (2)	0.68926 (15)	0.95176 (14)	0.0269 (5)	
H20	0.4656	0.7198	0.9588	0.032*	
C21	0.0916 (2)	1.04115 (15)	0.69334 (15)	0.0282 (5)	
H21A	0.0245	1.1008	0.7182	0.042*	
H21B	0.0476	1.0116	0.6524	0.042*	
H21C	0.1818	1.0606	0.6593	0.042*	
C22	−0.0155 (2)	0.92602 (16)	0.81716 (16)	0.0299 (5)	
H22A	0.0011	0.8803	0.8705	0.045*	
H22B	−0.045	0.8902	0.7734	0.045*	
H22C	−0.0921	0.9837	0.8365	0.045*	
C23	0.3361 (2)	1.06479 (16)	0.92679 (14)	0.0274 (5)	
H23A	0.2466	1.1054	0.9587	0.041*	
H23B	0.4042	1.1089	0.8981	0.041*	
H23C	0.3795	1.0157	0.9705	0.041*	
C24	0.3249 (2)	0.78089 (14)	0.58056 (13)	0.0191 (4)	
H24	0.3926	0.8297	0.5659	0.023*	
C25	0.2548 (2)	0.78407 (15)	0.49501 (13)	0.0213 (4)	
C26	0.3765 (2)	0.74420 (15)	0.41478 (13)	0.0224 (4)	
H26	0.4423	0.7939	0.3956	0.027*	
C27	0.4645 (2)	0.64260 (15)	0.43479 (14)	0.0269 (5)	
H27A	0.5411	0.623	0.3809	0.032*	
H27B	0.4018	0.5915	0.4485	0.032*	
C28	0.5328 (2)	0.64693 (15)	0.51624 (14)	0.0248 (4)	
H28A	0.5916	0.5802	0.5296	0.03*	
H28B	0.5975	0.6967	0.5017	0.03*	
C29	0.4158 (2)	0.67659 (14)	0.59900 (13)	0.0208 (4)	
H29	0.3513	0.6257	0.6133	0.025*	
O30	0.30900 (16)	0.73471 (10)	0.33928 (9)	0.0268 (3)	
C31	0.3358 (2)	0.79233 (15)	0.26237 (13)	0.0223 (4)	
O32	0.40856 (17)	0.85680 (11)	0.25168 (10)	0.0316 (4)	
C33	0.2631 (2)	0.76877 (15)	0.19191 (13)	0.0208 (4)	
C34	0.2657 (2)	0.83032 (15)	0.11194 (13)	0.0247 (5)	
H34	0.3158	0.8849	0.1024	0.03*	
C35	0.1955 (2)	0.81243 (17)	0.04616 (14)	0.0286 (5)	
H35	0.1968	0.855	−0.0084	0.034*	
C36	0.1237 (2)	0.73291 (18)	0.05975 (14)	0.0330 (5)	
H36	0.075	0.7209	0.0147	0.04*	
C37	0.1222 (3)	0.67046 (18)	0.13876 (15)	0.0344 (5)	
H37	0.0732	0.6153	0.1476	0.041*	
C38	0.1919 (2)	0.68816 (16)	0.20503 (14)	0.0296 (5)	
H38	0.1909	0.6452	0.2593	0.036*	
C39	0.1840 (2)	0.89252 (15)	0.46698 (14)	0.0253 (5)	
H39A	0.1477	0.8901	0.4107	0.03*	
H39B	0.0986	0.9141	0.5153	0.03*	
C40	0.2753 (2)	0.97374 (15)	0.44990 (15)	0.0303 (5)	
H40A	0.3069	0.982	0.5066	0.036*	
H40B	0.3627	0.954	0.4023	0.036*	
O41	0.1917 (2)	1.06636 (12)	0.42091 (11)	0.0461 (5)	
H41	0.2305	1.0848	0.3679	0.069*	
O42	0.47536 (14)	0.68229 (10)	0.67742 (9)	0.0223 (3)	
C43	0.5313 (3)	0.58881 (17)	0.71882 (16)	0.0364 (6)	
H43A	0.5402	0.599	0.7814	0.044*	
H43B	0.4618	0.5432	0.724	0.044*	
O44	0.6660 (2)	0.54241 (13)	0.67142 (12)	0.0538 (5)	
C45	0.7765 (3)	0.5978 (3)	0.6691 (3)	0.0826 (13)	
H45A	0.7436	0.6683	0.6492	0.124*	
H45B	0.8639	0.5691	0.6262	0.124*	
H45C	0.7983	0.5943	0.7301	0.124*	
C46	0.1359 (2)	0.72061 (16)	0.51442 (14)	0.0273 (5)	
H46A	0.096	0.7225	0.4595	0.041*	
H46B	0.0588	0.748	0.5644	0.041*	
H46C	0.1771	0.6508	0.5318	0.041*	
C47	0.1268 (2)	0.40365 (17)	0.63928 (16)	0.0345 (5)	
H47	0.0799	0.4303	0.59	0.041*	
C48	0.1481 (2)	0.46772 (17)	0.69805 (16)	0.0343 (5)	
H48	0.1157	0.5383	0.6892	0.041*	
C49	0.2161 (2)	0.42938 (16)	0.76949 (16)	0.0335 (5)	
H49	0.2298	0.4736	0.8102	0.04*	
C50	0.2644 (3)	0.32710 (17)	0.78217 (16)	0.0356 (5)	
H50	0.3119	0.3008	0.8312	0.043*	
C51	0.2433 (3)	0.26278 (17)	0.72304 (16)	0.0350 (5)	
H51	0.2769	0.1923	0.7314	0.042*	
C52	0.1735 (2)	0.30103 (17)	0.65192 (16)	0.0329 (5)	
H52	0.1578	0.2569	0.6119	0.04*	

### Atomic displacement parameters (Å^2^)

**Table:**

	*U*^11^	*U*^22^	*U*^33^	*U*^12^	*U*^13^	*U*^23^
O1	0.0219 (7)	0.0173 (7)	0.0177 (7)	−0.0039 (5)	−0.0048 (5)	0.0020 (5)
C2	0.0178 (10)	0.0194 (10)	0.0209 (10)	−0.0022 (8)	−0.0001 (8)	−0.0021 (8)
O3	0.0242 (7)	0.0203 (7)	0.0186 (7)	−0.0082 (6)	−0.0052 (6)	0.0023 (5)
C4	0.0169 (10)	0.0187 (10)	0.0202 (10)	−0.0046 (7)	−0.0050 (8)	0.0029 (8)
C5	0.0185 (10)	0.0174 (9)	0.0168 (9)	−0.0019 (7)	−0.0045 (8)	0.0045 (8)
C6	0.0198 (10)	0.0191 (10)	0.0190 (10)	−0.0024 (8)	−0.0063 (8)	0.0030 (8)
C7	0.0214 (10)	0.0179 (10)	0.0223 (10)	−0.0037 (8)	−0.0089 (8)	0.0056 (8)
C8	0.0252 (11)	0.0160 (9)	0.0202 (10)	−0.0040 (8)	−0.0065 (8)	0.0021 (8)
C9	0.0240 (11)	0.0171 (10)	0.0229 (10)	−0.0012 (8)	−0.0040 (8)	−0.0005 (8)
C10	0.0165 (10)	0.0198 (10)	0.0255 (10)	−0.0021 (8)	−0.0060 (8)	−0.0011 (8)
O11	0.0350 (8)	0.0217 (7)	0.0201 (7)	−0.0099 (6)	−0.0021 (6)	0.0025 (6)
O12	0.0216 (7)	0.0199 (7)	0.0212 (7)	−0.0036 (6)	−0.0088 (6)	0.0026 (6)
C13	0.0185 (10)	0.0228 (10)	0.0232 (10)	−0.0073 (8)	−0.0083 (8)	−0.0012 (8)
O14	0.0221 (8)	0.0314 (8)	0.0292 (8)	−0.0074 (6)	−0.0062 (6)	0.0090 (6)
C15	0.0234 (11)	0.0225 (10)	0.0220 (10)	−0.0046 (8)	−0.0083 (8)	0.0000 (8)
C16	0.0227 (11)	0.0294 (11)	0.0286 (11)	−0.0074 (9)	−0.0074 (9)	0.0006 (9)
C17	0.0279 (12)	0.0309 (12)	0.0389 (13)	−0.0003 (9)	−0.0163 (10)	0.0041 (10)
C18	0.0432 (14)	0.0257 (11)	0.0294 (12)	−0.0017 (10)	−0.0157 (10)	0.0064 (9)
C19	0.0397 (13)	0.0277 (12)	0.0258 (11)	−0.0066 (10)	−0.0023 (10)	0.0034 (9)
C20	0.0264 (11)	0.0275 (11)	0.0248 (11)	−0.0033 (9)	−0.0046 (9)	0.0022 (9)
C21	0.0295 (11)	0.0220 (11)	0.0359 (12)	0.0001 (9)	−0.0170 (10)	−0.0024 (9)
C22	0.0181 (11)	0.0296 (12)	0.0413 (13)	−0.0026 (9)	−0.0032 (9)	−0.0092 (10)
C23	0.0284 (11)	0.0287 (11)	0.0265 (11)	−0.0050 (9)	−0.0080 (9)	−0.0042 (9)
C24	0.0206 (10)	0.0190 (10)	0.0188 (10)	−0.0053 (8)	−0.0060 (8)	0.0013 (8)
C25	0.0249 (11)	0.0238 (10)	0.0171 (10)	−0.0059 (8)	−0.0087 (8)	0.0022 (8)
C26	0.0293 (11)	0.0230 (10)	0.0180 (10)	−0.0079 (8)	−0.0093 (8)	0.0001 (8)
C27	0.0346 (12)	0.0238 (11)	0.0212 (10)	−0.0040 (9)	−0.0041 (9)	−0.0024 (9)
C28	0.0275 (11)	0.0201 (10)	0.0248 (11)	0.0016 (8)	−0.0056 (9)	−0.0038 (8)
C29	0.0228 (10)	0.0209 (10)	0.0195 (10)	−0.0027 (8)	−0.0083 (8)	0.0003 (8)
O30	0.0391 (9)	0.0297 (8)	0.0164 (7)	−0.0147 (7)	−0.0100 (6)	0.0027 (6)
C31	0.0229 (10)	0.0226 (10)	0.0191 (10)	−0.0027 (8)	−0.0018 (8)	0.0011 (8)
O32	0.0425 (9)	0.0319 (8)	0.0260 (8)	−0.0188 (7)	−0.0114 (7)	0.0054 (6)
C33	0.0200 (10)	0.0236 (10)	0.0165 (10)	−0.0013 (8)	−0.0003 (8)	−0.0039 (8)
C34	0.0237 (11)	0.0251 (11)	0.0227 (10)	−0.0014 (8)	−0.0022 (8)	−0.0013 (9)
C35	0.0271 (11)	0.0364 (12)	0.0180 (10)	0.0023 (9)	−0.0036 (9)	−0.0017 (9)
C36	0.0323 (12)	0.0495 (14)	0.0196 (11)	−0.0069 (10)	−0.0068 (9)	−0.0109 (10)
C37	0.0412 (13)	0.0407 (13)	0.0272 (12)	−0.0188 (11)	−0.0063 (10)	−0.0072 (10)
C38	0.0351 (12)	0.0334 (12)	0.0218 (11)	−0.0120 (10)	−0.0039 (9)	−0.0012 (9)
C39	0.0263 (11)	0.0296 (11)	0.0181 (10)	0.0001 (9)	−0.0077 (8)	0.0029 (8)
C40	0.0350 (12)	0.0222 (11)	0.0282 (11)	0.0008 (9)	−0.0038 (9)	0.0043 (9)
O41	0.0580 (11)	0.0276 (9)	0.0340 (9)	0.0095 (8)	0.0067 (8)	0.0132 (7)
O42	0.0253 (7)	0.0208 (7)	0.0203 (7)	0.0018 (6)	−0.0096 (6)	−0.0009 (6)
C43	0.0510 (15)	0.0277 (12)	0.0318 (12)	0.0042 (11)	−0.0239 (11)	0.0017 (10)
O44	0.0635 (12)	0.0453 (10)	0.0521 (11)	0.0305 (9)	−0.0381 (10)	−0.0268 (9)
C45	0.0367 (17)	0.125 (3)	0.092 (3)	0.0260 (18)	−0.0341 (17)	−0.067 (2)
C46	0.0288 (11)	0.0358 (12)	0.0211 (10)	−0.0119 (9)	−0.0099 (9)	0.0026 (9)
C47	0.0288 (12)	0.0347 (13)	0.0369 (13)	−0.0037 (10)	−0.0053 (10)	0.0033 (10)
C48	0.0286 (12)	0.0248 (11)	0.0432 (14)	−0.0022 (9)	0.0024 (10)	0.0006 (10)
C49	0.0366 (13)	0.0272 (12)	0.0351 (13)	−0.0088 (10)	0.0008 (10)	−0.0063 (10)
C50	0.0390 (13)	0.0334 (13)	0.0345 (13)	−0.0102 (10)	−0.0070 (10)	0.0028 (10)
C51	0.0368 (13)	0.0249 (12)	0.0415 (14)	−0.0055 (10)	−0.0063 (11)	0.0020 (10)
C52	0.0337 (13)	0.0287 (12)	0.0365 (13)	−0.0090 (10)	−0.0034 (10)	−0.0043 (10)

### Geometric parameters (Å, º)

**Table:**

O1—C2	1.334 (2)	C27—C28	1.523 (3)
O1—C5	1.461 (2)	C27—H27A	0.99
C2—O11	1.189 (2)	C27—H27B	0.99
C2—O3	1.342 (2)	C28—C29	1.516 (3)
O3—C4	1.446 (2)	C28—H28A	0.99
C4—C24	1.544 (3)	C28—H28B	0.99
C4—C5	1.566 (3)	C29—O42	1.435 (2)
C4—H4	1.0	C29—H29	1.0
C5—C6	1.517 (3)	O30—C31	1.333 (2)
C5—C10	1.550 (3)	C31—O32	1.209 (2)
C6—C7	1.524 (3)	C31—C33	1.486 (3)
C6—H6A	0.99	C33—C38	1.385 (3)
C6—H6B	0.99	C33—C34	1.387 (3)
C7—O12	1.464 (2)	C34—C35	1.381 (3)
C7—C8	1.501 (3)	C34—H34	0.95
C7—H7	1.0	C35—C36	1.375 (3)
C8—C9	1.324 (3)	C35—H35	0.95
C8—C23	1.507 (3)	C36—C37	1.382 (3)
C9—C10	1.513 (3)	C36—H36	0.95
C9—H9	0.95	C37—C38	1.383 (3)
C10—C22	1.534 (3)	C37—H37	0.95
C10—C21	1.538 (3)	C38—H38	0.95
O12—C13	1.338 (2)	C39—C40	1.519 (3)
C13—O14	1.213 (2)	C39—H39A	0.99
C13—C15	1.485 (3)	C39—H39B	0.99
C15—C20	1.388 (3)	C40—O41	1.427 (3)
C15—C16	1.393 (3)	C40—H40A	0.99
C16—C17	1.383 (3)	C40—H40B	0.99
C16—H16	0.95	O41—H41	0.84
C17—C18	1.385 (3)	O42—C43	1.408 (2)
C17—H17	0.95	C43—O44	1.396 (3)
C18—C19	1.381 (3)	C43—H43A	0.99
C18—H18	0.95	C43—H43B	0.99
C19—C20	1.385 (3)	O44—C45	1.420 (4)
C19—H19	0.95	C45—H45A	0.98
C20—H20	0.95	C45—H45B	0.98
C21—H21A	0.98	C45—H45C	0.98
C21—H21B	0.98	C46—H46A	0.98
C21—H21C	0.98	C46—H46B	0.98
C22—H22A	0.98	C46—H46C	0.98
C22—H22B	0.98	C47—C52	1.380 (3)
C22—H22C	0.98	C47—C48	1.380 (3)
C23—H23A	0.98	C47—H47	0.95
C23—H23B	0.98	C48—C49	1.378 (3)
C23—H23C	0.98	C48—H48	0.95
C24—C29	1.536 (3)	C49—C50	1.378 (3)
C24—C25	1.571 (2)	C49—H49	0.95
C24—H24	1.0	C50—C51	1.386 (3)
C25—C46	1.535 (3)	C50—H50	0.95
C25—C26	1.547 (3)	C51—C52	1.383 (3)
C25—C39	1.545 (3)	C51—H51	0.95
C26—O30	1.455 (2)	C52—H52	0.95
C26—C27	1.500 (3)	C9—C40	5.831 (3)
C26—H26	1.0		
			
C2—O1—C5	110.64 (14)	C27—C26—C25	114.64 (16)
O11—C2—O1	124.53 (18)	O30—C26—H26	109.1
O11—C2—O3	123.74 (17)	C27—C26—H26	109.1
O1—C2—O3	111.71 (16)	C25—C26—H26	109.1
C2—O3—C4	110.32 (14)	C26—C27—C28	109.18 (16)
O3—C4—C24	109.71 (15)	C26—C27—H27A	109.8
O3—C4—C5	102.47 (14)	C28—C27—H27A	109.8
C24—C4—C5	120.64 (15)	C26—C27—H27B	109.8
O3—C4—H4	107.8	C28—C27—H27B	109.8
C24—C4—H4	107.8	H27A—C27—H27B	108.3
C5—C4—H4	107.8	C29—C28—C27	110.00 (16)
O1—C5—C6	106.35 (14)	C29—C28—H28A	109.7
O1—C5—C10	107.29 (14)	C27—C28—H28A	109.7
C6—C5—C10	110.80 (15)	C29—C28—H28B	109.7
O1—C5—C4	101.92 (14)	C27—C28—H28B	109.7
C6—C5—C4	117.25 (15)	H28A—C28—H28B	108.2
C10—C5—C4	112.21 (15)	O42—C29—C28	111.95 (15)
C5—C6—C7	112.96 (16)	O42—C29—C24	106.93 (15)
C5—C6—H6A	109.0	C28—C29—C24	110.66 (16)
C7—C6—H6A	109.0	O42—C29—H29	109.1
C5—C6—H6B	109.0	C28—C29—H29	109.1
C7—C6—H6B	109.0	C24—C29—H29	109.1
H6A—C6—H6B	107.8	C31—O30—C26	119.33 (15)
O12—C7—C8	105.62 (15)	O32—C31—O30	124.21 (18)
O12—C7—C6	110.35 (14)	O32—C31—C33	124.19 (18)
C8—C7—C6	114.15 (16)	O30—C31—C33	111.59 (16)
O12—C7—H7	108.9	C38—C33—C34	119.78 (19)
C8—C7—H7	108.9	C38—C33—C31	121.56 (18)
C6—C7—H7	108.9	C34—C33—C31	118.65 (18)
C9—C8—C7	120.99 (18)	C35—C34—C33	120.20 (19)
C9—C8—C23	122.92 (19)	C35—C34—H34	119.9
C7—C8—C23	116.07 (17)	C33—C34—H34	119.9
C8—C9—C10	126.79 (18)	C36—C35—C34	119.9 (2)
C8—C9—H9	116.6	C36—C35—H35	120.1
C10—C9—H9	116.6	C34—C35—H35	120.1
C9—C10—C22	108.36 (17)	C35—C36—C37	120.3 (2)
C9—C10—C21	108.05 (16)	C35—C36—H36	119.9
C22—C10—C21	108.13 (16)	C37—C36—H36	119.9
C9—C10—C5	109.25 (15)	C38—C37—C36	120.1 (2)
C22—C10—C5	111.47 (16)	C38—C37—H37	119.9
C21—C10—C5	111.47 (16)	C36—C37—H37	119.9
C13—O12—C7	117.11 (14)	C37—C38—C33	119.7 (2)
O14—C13—O12	123.85 (17)	C37—C38—H38	120.1
O14—C13—C15	124.22 (18)	C33—C38—H38	120.1
O12—C13—C15	111.92 (16)	C40—C39—C25	118.35 (17)
C20—C15—C16	119.80 (18)	C40—C39—H39A	107.7
C20—C15—C13	121.31 (18)	C25—C39—H39A	107.7
C16—C15—C13	118.79 (18)	C40—C39—H39B	107.7
C17—C16—C15	120.06 (19)	C25—C39—H39B	107.7
C17—C16—H16	120.0	H39A—C39—H39B	107.1
C15—C16—H16	120.0	O41—C40—C39	109.16 (18)
C18—C17—C16	119.9 (2)	O41—C40—H40A	109.8
C18—C17—H17	120.0	C39—C40—H40A	109.8
C16—C17—H17	120.0	O41—C40—H40B	109.8
C19—C18—C17	120.1 (2)	C39—C40—H40B	109.8
C19—C18—H18	120.0	H40A—C40—H40B	108.3
C17—C18—H18	120.0	C40—O41—H41	109.5
C18—C19—C20	120.4 (2)	C43—O42—C29	115.15 (15)
C18—C19—H19	119.8	O44—C43—O42	113.8 (2)
C20—C19—H19	119.8	O44—C43—H43A	108.8
C15—C20—C19	119.7 (2)	O42—C43—H43A	108.8
C15—C20—H20	120.1	O44—C43—H43B	108.8
C19—C20—H20	120.1	O42—C43—H43B	108.8
C10—C21—H21A	109.5	H43A—C43—H43B	107.7
C10—C21—H21B	109.5	C43—O44—C45	112.71 (19)
H21A—C21—H21B	109.5	O44—C45—H45A	109.5
C10—C21—H21C	109.5	O44—C45—H45B	109.5
H21A—C21—H21C	109.5	H45A—C45—H45B	109.5
H21B—C21—H21C	109.5	O44—C45—H45C	109.5
C10—C22—H22A	109.5	H45A—C45—H45C	109.5
C10—C22—H22B	109.5	H45B—C45—H45C	109.5
H22A—C22—H22B	109.5	C25—C46—H46A	109.5
C10—C22—H22C	109.5	C25—C46—H46B	109.5
H22A—C22—H22C	109.5	H46A—C46—H46B	109.5
H22B—C22—H22C	109.5	C25—C46—H46C	109.5
C8—C23—H23A	109.5	H46A—C46—H46C	109.5
C8—C23—H23B	109.5	H46B—C46—H46C	109.5
H23A—C23—H23B	109.5	C52—C47—C48	120.1 (2)
C8—C23—H23C	109.5	C52—C47—H47	119.9
H23A—C23—H23C	109.5	C48—C47—H47	119.9
H23B—C23—H23C	109.5	C49—C48—C47	120.1 (2)
C29—C24—C4	112.45 (15)	C49—C48—H48	120.0
C29—C24—C25	111.56 (15)	C47—C48—H48	120.0
C4—C24—C25	111.32 (15)	C48—C49—C50	120.2 (2)
C29—C24—H24	107.1	C48—C49—H49	119.9
C4—C24—H24	107.1	C50—C49—H49	119.9
C25—C24—H24	107.1	C49—C50—C51	119.7 (2)
C46—C25—C26	110.71 (16)	C49—C50—H50	120.2
C46—C25—C39	107.03 (16)	C51—C50—H50	120.2
C26—C25—C39	108.36 (15)	C52—C51—C50	120.2 (2)
C46—C25—C24	111.22 (15)	C52—C51—H51	119.9
C26—C25—C24	107.84 (15)	C50—C51—H51	119.9
C39—C25—C24	111.66 (15)	C47—C52—C51	119.7 (2)
O30—C26—C27	106.89 (15)	C47—C52—H52	120.2
O30—C26—C25	107.73 (15)	C51—C52—H52	120.2
			
C5—O1—C2—O11	−176.77 (18)	C5—C4—C24—C29	87.2 (2)
C5—O1—C2—O3	1.9 (2)	O3—C4—C24—C25	94.64 (17)
O11—C2—O3—C4	−171.43 (18)	C5—C4—C24—C25	−146.76 (16)
O1—C2—O3—C4	9.9 (2)	C29—C24—C25—C46	69.7 (2)
C2—O3—C4—C24	113.11 (16)	C4—C24—C25—C46	−56.9 (2)
C2—O3—C4—C5	−16.21 (18)	C29—C24—C25—C26	−51.9 (2)
C2—O1—C5—C6	−134.83 (16)	C4—C24—C25—C26	−178.43 (15)
C2—O1—C5—C10	106.55 (16)	C29—C24—C25—C39	−170.86 (16)
C2—O1—C5—C4	−11.50 (18)	C4—C24—C25—C39	62.6 (2)
O3—C4—C5—O1	16.00 (16)	C46—C25—C26—O30	50.78 (19)
C24—C4—C5—O1	−106.16 (17)	C39—C25—C26—O30	−66.31 (18)
O3—C4—C5—C6	131.61 (16)	C24—C25—C26—O30	172.66 (14)
C24—C4—C5—C6	9.4 (2)	C46—C25—C26—C27	−68.1 (2)
O3—C4—C5—C10	−98.47 (16)	C39—C25—C26—C27	174.86 (16)
C24—C4—C5—C10	139.36 (17)	C24—C25—C26—C27	53.8 (2)
O1—C5—C6—C7	−57.91 (19)	O30—C26—C27—C28	−177.59 (15)
C10—C5—C6—C7	58.4 (2)	C25—C26—C27—C28	−58.3 (2)
C4—C5—C6—C7	−171.06 (15)	C26—C27—C28—C29	59.7 (2)
C5—C6—C7—O12	81.26 (19)	C27—C28—C29—O42	−179.61 (15)
C5—C6—C7—C8	−37.5 (2)	C27—C28—C29—C24	−60.4 (2)
O12—C7—C8—C9	−114.50 (19)	C4—C24—C29—O42	−54.6 (2)
C6—C7—C8—C9	6.9 (3)	C25—C24—C29—O42	179.52 (14)
O12—C7—C8—C23	67.2 (2)	C4—C24—C29—C28	−176.75 (15)
C6—C7—C8—C23	−171.41 (16)	C25—C24—C29—C28	57.4 (2)
C7—C8—C9—C10	2.7 (3)	C27—C26—O30—C31	−120.95 (19)
C23—C8—C9—C10	−179.15 (18)	C25—C26—O30—C31	115.37 (18)
C8—C9—C10—C22	139.3 (2)	C26—O30—C31—O32	−3.6 (3)
C8—C9—C10—C21	−103.8 (2)	C26—O30—C31—C33	177.38 (16)
C8—C9—C10—C5	17.7 (3)	O32—C31—C33—C38	174.2 (2)
O1—C5—C10—C9	69.18 (18)	O30—C31—C33—C38	−6.9 (3)
C6—C5—C10—C9	−46.5 (2)	O32—C31—C33—C34	−6.7 (3)
C4—C5—C10—C9	−179.68 (15)	O30—C31—C33—C34	172.23 (17)
O1—C5—C10—C22	−50.6 (2)	C38—C33—C34—C35	1.1 (3)
C6—C5—C10—C22	−166.25 (16)	C31—C33—C34—C35	−178.07 (18)
C4—C5—C10—C22	60.6 (2)	C33—C34—C35—C36	−0.5 (3)
O1—C5—C10—C21	−171.49 (14)	C34—C35—C36—C37	−0.4 (3)
C6—C5—C10—C21	72.82 (19)	C35—C36—C37—C38	0.6 (3)
C4—C5—C10—C21	−60.3 (2)	C36—C37—C38—C33	0.0 (3)
C8—C7—O12—C13	−158.97 (15)	C34—C33—C38—C37	−0.8 (3)
C6—C7—O12—C13	77.20 (19)	C31—C33—C38—C37	178.25 (19)
C7—O12—C13—O14	5.0 (3)	C46—C25—C39—C40	175.91 (17)
C7—O12—C13—C15	−173.77 (15)	C26—C25—C39—C40	−64.7 (2)
O14—C13—C15—C20	−163.81 (19)	C24—C25—C39—C40	54.0 (2)
O12—C13—C15—C20	15.0 (3)	C25—C39—C40—O41	177.32 (17)
O14—C13—C15—C16	12.6 (3)	C28—C29—O42—C43	−74.0 (2)
O12—C13—C15—C16	−168.65 (17)	C24—C29—O42—C43	164.63 (17)
C20—C15—C16—C17	0.5 (3)	C29—O42—C43—O44	76.5 (2)
C13—C15—C16—C17	−175.95 (19)	O42—C43—O44—C45	64.8 (3)
C15—C16—C17—C18	0.5 (3)	C52—C47—C48—C49	0.1 (3)
C16—C17—C18—C19	−1.3 (3)	C47—C48—C49—C50	0.6 (3)
C17—C18—C19—C20	1.2 (3)	C48—C49—C50—C51	−0.4 (3)
C16—C15—C20—C19	−0.6 (3)	C49—C50—C51—C52	−0.3 (3)
C13—C15—C20—C19	175.70 (19)	C48—C47—C52—C51	−0.8 (3)
C18—C19—C20—C15	−0.2 (3)	C50—C51—C52—C47	0.9 (3)
O3—C4—C24—C29	−31.4 (2)		

### Hydrogen-bond geometry (Å, º)

*Cg* is the centroid of the C47–C52 ring.

**Table:**

*D*—H···*A*	*D*—H	H···*A*	*D*···*A*	*D*—H···*A*
C6—H6*B*···O42	0.99	2.32	3.095 (2)	135
C28—H28*A*···O44	0.99	2.37	2.989 (3)	120
O41—H41···O14^i^	0.84	2.06	2.888 (2)	170
C7—H7···O32^i^	1.00	2.34	3.269 (2)	155
C18—H18···O11^ii^	0.95	2.53	3.465 (2)	168
C49—H49···O11	0.95	2.46	3.300 (3)	147
C27—H27*A*···*Cg*^iii^	0.95	2.64	3.514 (2)	147

Symmetry codes: (i) −*x*+1, −*y*+2, −*z*+1; (ii) −*x*+1, −*y*+1, −*z*+2; (iii) −*x*+1, −*y*+1, −*z*+1.
